# Supplementary figures and images for: Grain Quality Characterization of Hybrid Rice Restorer Lines with Resilience to Suboptimal Temperatures during Filling Stage
Source: Foods. 2022 Nov 4;11(21):3513. doi: 10.3390/foods11213513 (PMC9658161; doi:10.3390/foods11213513)

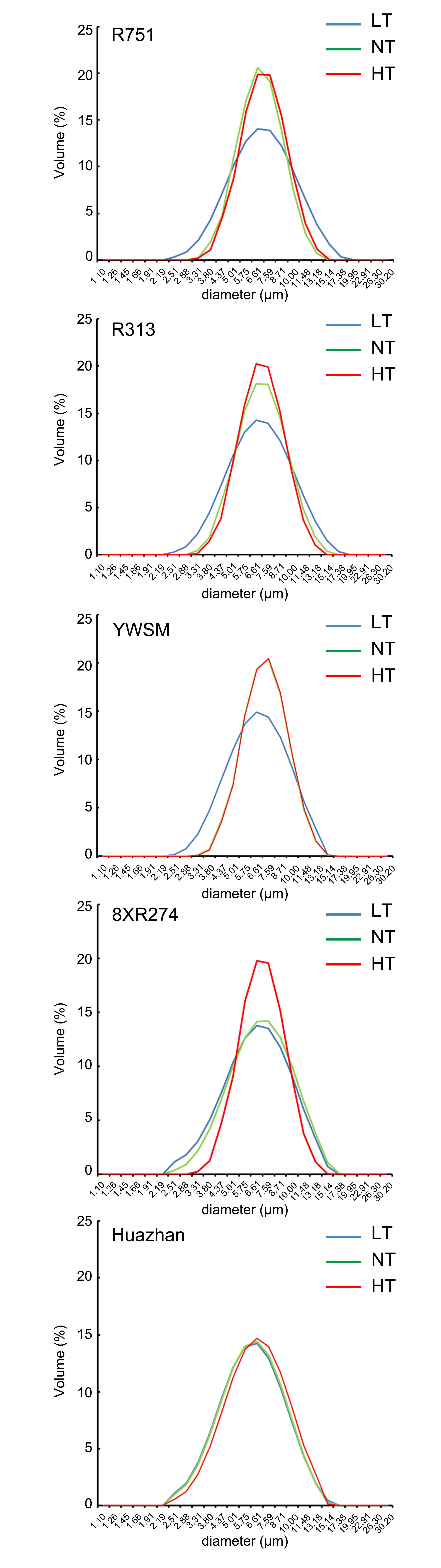

Supplement: Supplementary file 1 [file foods-11-03513-s001.zip › Supplementary Fig.1 granule size distribution.tif]
